# Transcatheter aortic valve replacement using the two-step inflation technique and the kissing-balloon technique for a patient with a protruding stent in the left main coronary artery: a case report

**DOI:** 10.1093/ehjcr/ytad575

**Published:** 2023-11-30

**Authors:** Takuma Ohi, Naoki Watanabe, Kensuke Takagi, Itsuro Morishima

**Affiliations:** Department of Cardiology, Ogaki Municipal Hospital, 4-86, Minaminokawa-cho, Ogaki 503-8502, Japan; Department of Cardiology, Ogaki Municipal Hospital, 4-86, Minaminokawa-cho, Ogaki 503-8502, Japan; Department of Cardiovascular Medicine, National Cerebral and Cardiovascular Center, Suita, Japan; Department of Cardiology, Ogaki Municipal Hospital, 4-86, Minaminokawa-cho, Ogaki 503-8502, Japan

**Keywords:** Transcatheter aortic valve replacement, Left main coronary artery, Protruding stent, Two-step inflation technique, Kissing-balloon technique, Case report

## Abstract

**Background:**

When performing transcatheter aortic valve replacement (TAVR) for a patient with a protruding stent in the coronary arteries, there is a risk of stent deformation and coronary occlusion. However, safe and optimal methods have not been established.

**Case summary:**

An 87-year-old woman with a protruding stent in the left main coronary artery (LMCA) underwent TAVR. The two-step inflation and kissing-balloon techniques were performed to optimize the transcatheter heart valve (THV) and to avoid LMCA stent deformation. The THV was implemented with minimal aortic regurgitation and no deformation of the stent in the LMCA.

**Discussion:**

This was the first case report of TAVR, performed in a patient with a protruding stent in the coronary arteries, using the kissing-balloon technique and the two-step inflation technique. The combination of these two techniques was the optimal method for THV implantation without stent deformation in coronary arteries.

**Learning points**

- The combination of the two-step inflation technique and the kissing-balloon technique optimized the transcatheter heart valve (THV) without stent deformation in transcatheter aortic valve replacement (TAVR) for a patient with a protruding stent in a coronary artery.
- When we use the kissing-balloon technique in the TAVR procedure, we can decide how large we expand a deployment balloon of a balloon-expandable THV based on the widely used calculation methods for determining balloon size in the kissing-balloon technique.

## Introduction

Among patients with a protruding stent from the ostium of the coronary artery, transcatheter aortic valve replacement (TAVR) bears the risk of stent deformation and coronary occlusion. Although there was a report in which TAVR was performed for such a case using the kissing-balloon technique,^1^ safe and optimal methods for these cases have not been established. This study reports a successful TAVR procedure that combined the two-step inflation technique and the kissing-balloon technique to optimize transcatheter heart valve (THV) positioning without stent deformation in the left main coronary artery (LMCA).

## Summary figure

**Figure:**
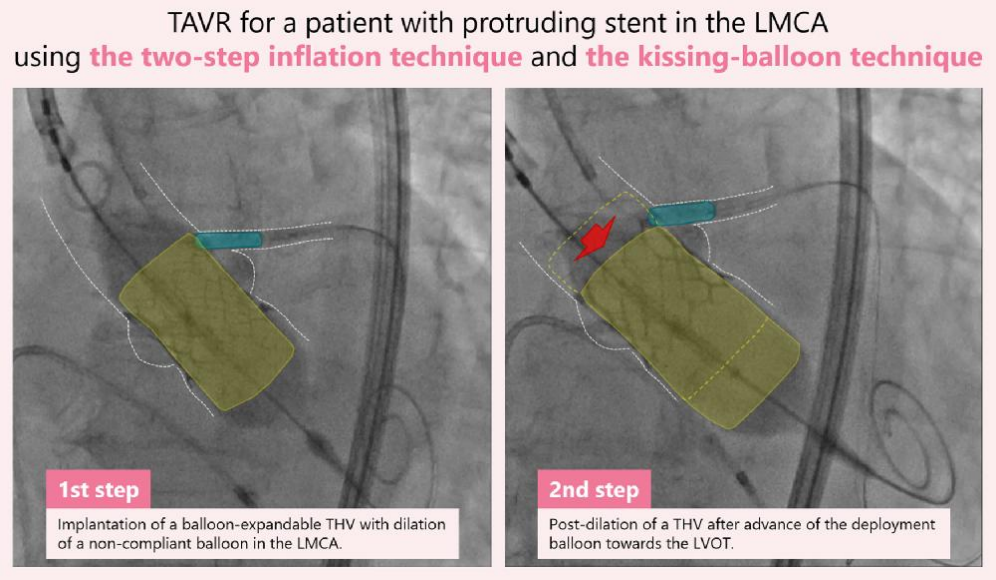


## Case presentation

An 87-year-old woman, whose past medical history was acute cholecystitis and hepatocellular carcinoma, underwent percutaneous coronary intervention (PCI) for acute myocardial infarction with a 3.0 × 28 mm drug-eluting stent (Xience Skypoint^®^; Abbott Laboratories, IL, USA), implanted in the LMCA. Concurrently, physical examination revealed a harsh systolic murmur at the aortic valve area. While transthoracic echocardiography showed an aortic root dimension of 26.5 mm, a left atrial dimension of 47.1 mm, a left ventricular end-diastolic dimension of 40.4 mm, a left ventricular end-systolic dimension of 27.8 mm, a left ventricular ejection fraction of 48.9%, and severe hypokinesis from the anteroseptal to the apex, it also revealed severe aortic stenosis evidenced by an aortic valve area of 0.29 cm^2^, peak velocity of 4.40 m/s, and a mean pressure gradient of 50.2 mmHg (see Supplementary material online, *Video S1*). Two months post-discharge, she presented with chest pain and syncope. Physical examination revealed haemodynamically stable with a heart rate of 68 b.p.m., blood pressure of 121/69 mmHg, respiratory rate of 16 breaths/min, and oxygen saturation of 97%. The electrocardiogram showed no bradyarrhythmia causing syncope or no ST-segment change indicating stent thrombosis, so we judged that aortic stenosis had caused these symptoms and decided to perform TAVR.

**Figure 1:**
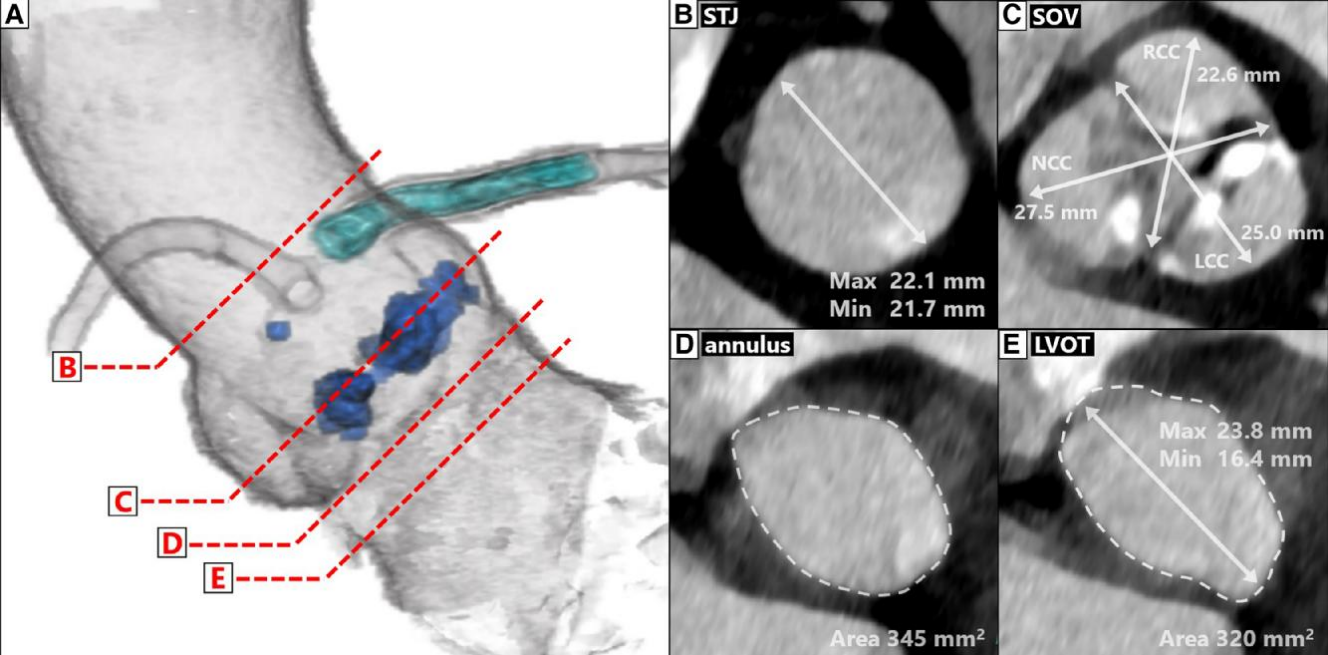
The aortic valve complex as seen on the preoperative computed tomography. In the perpendicular view (*A*), sections B–E showed the sinotubular junction (*B*), the sinus of Valsalva (*C*), the aortic annulus (*D*), and the left ventricular outflow tract (*E*), respectively. CT, computed tomography; STJ, sinotubular junction; SOV, sinus of Valsalva; LVOT, left ventricular outflow tract; RCC, right coronary cusp; LCC, left coronary cusp; NCC, non-coronary cusp.

**Figure 2:**
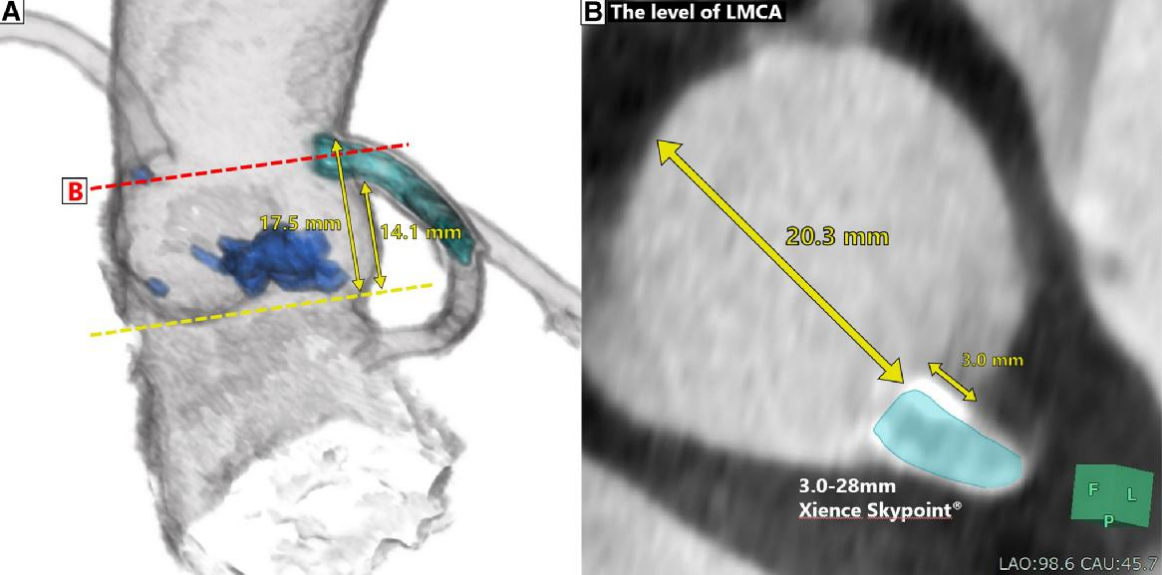
The preoperative computed tomography showed the height of the left main coronary artery from the annulus plane (*A*) and at the level of the left main coronary artery, there was a 3.0 mm protrusion of the left main coronary artery stent from the ostium with a distance of 20.3 mm to the opposite vessel wall was shown (*B*). CT, computed tomography; LMCA, left main coronary artery.

Computed tomography (CT) scans revealed a tricuspid aortic valve with an aortic annulus area of 345 mm^2^ and a perimeter of 67.0 mm. The diameter of the sinus of Valsalva measured 22.6 × 25.0 × 27.5 mm. The diameter of the sinotubular junction (STJ) measured 21.7 × 22.1 mm. Meanwhile, the diameter of the left ventricular outflow tract (LVOT) was 16.4 × 23.8 mm, and it had an area of 320 mm^2^. Minimal calcification was observed in the STJ and LVOT (*Figure 1*). The right and left coronary heights from the annular plane were 14.4–16.5 and 14.1–17.5 mm (*Figure 2A*), respectively. The CT also showed a 3.0 mm protrusion of the LMCA stent from the ostium with a distance of 20.3 mm to the opposite vessel wall (*Figure 2B*). The access routes from the right and left femoral arteries were unobstructed. Based on these measured values, both a 23 mm balloon-expandable THV and a 26 mm self-expandable THV were considered suitable options. However, when considering postprocedural coronary events and coronary access, a balloon-expandable THV was the preferred choice. Additionally, given that we anticipated that the frames of a 26 mm self-expandable THV would definitely come in contact with the LMCA stent, potentially cause stenting fracture and flow limitation, a 23 mm balloon-expandable THV was finally chosen (SAPIEN 3^®^; Edwards Lifesciences, CA, USA). However, the potential THV contact and deformation of the LMCA stent prompted the combined use of the two-step inflation technique and the kissing-balloon technique during THV implantation.

**Figure 3:**
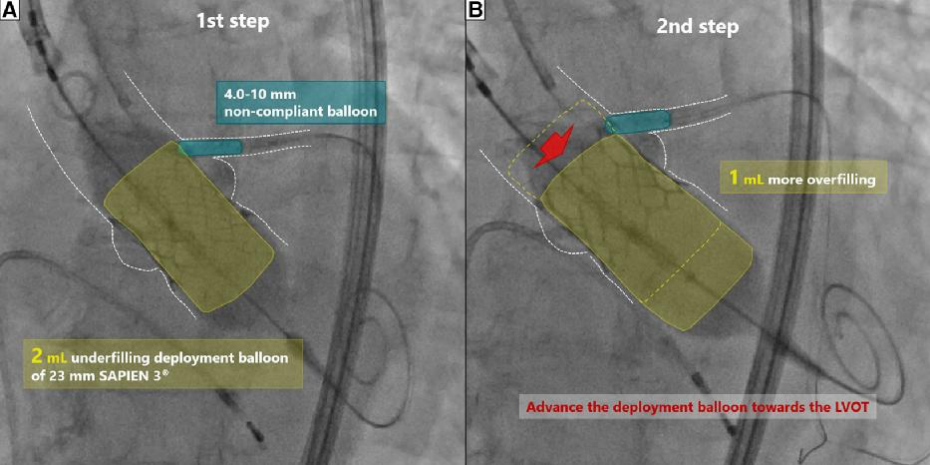
The two-step inflation technique and the kissing-balloon technique: first, the prosthetic valve was implanted with the 2 mL underfilling deployment balloon while inflating the coronary balloon in the stent in the LMCA (*A*). Second, the deployment balloon was dilated on the side of the LVOT by 1 mL more overfilling while inflating the coronary balloon (*B*). LMCA, left main coronary artery; LVOT, left ventricular outflow tract.

**Figure 4:**
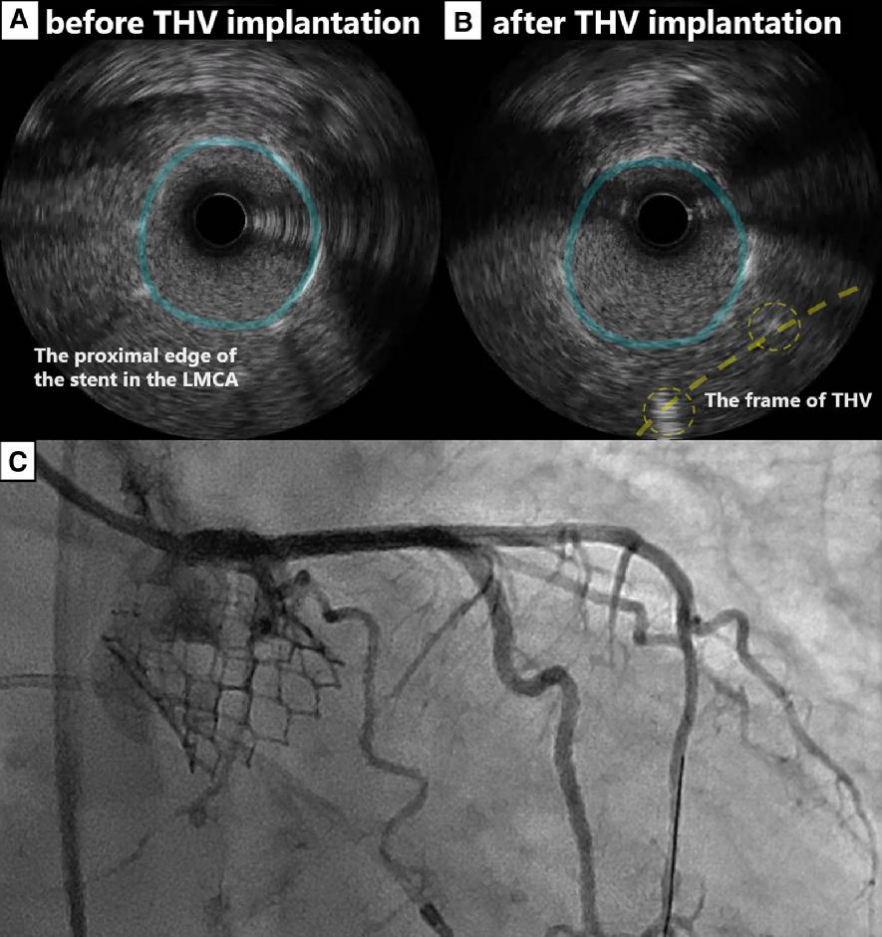
The intravascular ultrasound findings of the ostium of the stent in the left main coronary artery before (*A*) and after (*B*) the transcatheter heart valve was implanted, and coronary angiography (*C*): the stent (solid lines) in the left main coronary artery was not deformed. In (*B*), the frame of the transcatheter heart valve (dashed lines) was proximal to the stent in the left main coronary artery. IVUS, intravascular ultrasound, LMCA, left main coronary artery, THV, transcatheter heart valve.

**Figure 5:**
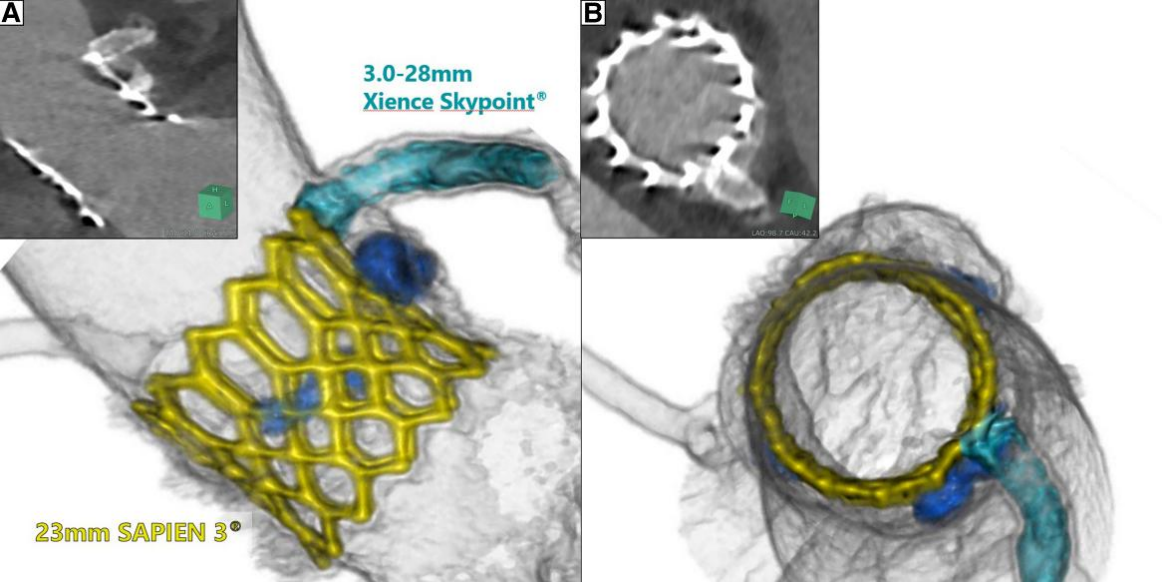
The transcatheter heart valve and the stent in the left main coronary artery on computed tomography 3 days after transcatheter aortic valve replacement. THV, transcatheter heart valve; LMCA, left main coronary artery; CT, computed tomography; TAVR, transcatheter aortic valve replacement.

Transcatheter aortic valve replacement was performed via percutaneous transfemoral approach under monitored anaesthesia care. A guiding catheter was delivered towards the LMCA via the left radial approach, and a coronary guiding wire was inserted through the LMCA to the left anterior descending artery. We implanted the THV 2 mL underfilled in the first step while dilating a 4.0 × 10 mm non-compliant coronary balloon (NC Kamui^®^; ASAHI INTECC, Aichi, Japan) in the LMCA stent (*Figure 3A* and Supplementary material online, *Video S2*). Secondly, the balloon was deployed slightly towards the LVOT, and it was dilated 1 mL underfilled. The coronary balloon was also inflated (*Figure 3B* and Supplementary material online, *Video S3*). Angiography and echocardiography showed improved THV expansion and reduced aortic regurgitation without compromised coronary flow. Intravascular ultrasound before and after THV implantation demonstrated an undistorted stent and THV implantation near the LMCA stent (*Figure 4*), while CT 3 days after TAVR also showed similar findings (*Figure 5*). Haemodynamics were consistently stable before and after THV implantation. After the second step of balloon dilation, left ventricular pressure was 167/21 mmHg and aortic pressure was 160/68 mmHg, indicating that the pressure gradient had disappeared. Postoperative echocardiography also revealed good THV mobility and improvement in aortic stenosis with a mean pressure gradient of 12.8 mmHg and an effective orifice area of 1.75 cm^2^ (see Supplementary material online, *Video S1*). Her postoperative course was uneventful.

## Discussion

There have been few cases on the application of TAVR in the treatment of patients with protruding stents in the coronary arteries. A previous report documented the application of the kissing-balloon technique in a patient with a protruding stent in the LMCA.^1^ This was the first case report to demonstrate a successful THV optimization and prevention of LMCA stent deformation, using a combination of the two-step inflation technique and the kissing-balloon technique.

Prior to performing TAVR, we meticulously consider various factors to determine the most appropriate type of THV suitable for the patient. In the present case, based on the measured values of the CT scan, either a 23 mm balloon-expandable THV or a 26 mm self-expandable THV could be considered optimal. The frame height of a 23 mm balloon-expandable THV is 18 mm; thus, we presumed it would interact with the LMCA stent. Contrastingly, the narrowest waist diameter of a 26 mm self-expandable THV is 22 mm and the distance from its bottom to this point is 26 mm. Even if we deployed it precisely to align with the level of the protruding stent in the LMCA, it would inevitably make contact with the stent unless the cell of its frame perfectly matched the LMCA stent, given that the distance from the ostium of the LMCA stent to the opposite side of the aortic wall was only 20.3 mm. In addition, employing the self-expandable THV would introduce uncertainties regarding the short- and long-term outcomes of the LMCA stent. For these reasons, we chose a 23 mm balloon-expandable THV for the treatment of this patient.

The two-step inflation technique is typically employed in TAVR for patients with narrow and calcified STJs to avoid aortic dissection or basal rupture and to optimize THV positioning.^2^ The usefulness of this technique for patients with protruding coronary stents is not widely known. However, it should be considered for such patients, except those at high risk of complete atrioventricular block, such as patients with right bundle branch block.

The kissing-balloon technique is commonly used in PCI for patients with bifurcation lesions to dilate the side branch without deforming the implanted stent in the main vessel. In the present case, the 23 mm balloon-expandable THV expanded by 2 mL underfilled and 1 mL underfilled, resulting in diameters of ∼21.6 and 22.3 mm, respectively. Given the 23.3 mm diameter of the vessel at the level of the LMCA ostium (*Figure 2A*) and the use of a 4.0 mm coronary balloon, the expected expansion of the deployment balloon was 22.9–25.1 mm, based on the widely used calculation methods for determining balloon size in the kissing-balloon technique (see Supplementary material online, *Figure S1*). Therefore, a 1–2 mL underfilled deployment balloon was considered safe. While the technique is not necessary for all TAVR patients with protruding coronary stents, it should be employed in cases, where the distance from the proximal edge of the coronary stent to the opposite vessel wall is less than the diameter of the THV and the coronary height from the annulus plane is less than the height of the THV.

## Supplementary Material

ytad575_Supplementary_Data

## Data Availability

The data underlying this article are available in this article and in its Supplementary material online.

## Lead author biography

Takuma Ohi graduated from Gifu university graduate school of medicine in 2017. He currently belongs to Department of Cardiology in Ogaki Municipal Hospital, Ogaki, Japan.

## Supplementary material

**Consent**: The authors confirmed that written consent for the submission and publication of this case report, including the images and associated text, was obtained from the patient in line with the COPE guidelines.

**Funding:** None declared.
