# Supplementary material for: Transcatheter aortic valve replacement using the two-step inflation technique and the kissing-balloon technique for a patient with a protruding stent in the left main coronary artery: a case report
Source: Eur Heart J Case Rep. 2023 Nov 30;7(12):ytad575. doi: 10.1093/ehjcr/ytad575 (PMC10711422; doi:10.1093/ehjcr/ytad575)
Supplement: ytad575_Supplementary_Data [file ytad575_supplementary_data.zip › figure and video legends of supplemantary material.docx]

**Figure and video legends of Supplementary material**:

**Figure S1**: Calculation methods which are widely used when they decide the balloon size using the kissing-balloon technique. Calculation method 1 and 2 are simplified formulas. D is the diameter of the proximal side of the main vessel, d_1_ is the diameter of the balloon for the main vessel, and d2 is the diameter of the balloon for the side branch, respectively. In calculation method 3, they sum the areas of the two semicircles whose diameter are d_1_ and d_2_ and the trapezium between them (S, blue oblique line). They assume the circle whose area is S and the diameter of the circle is D.

**Video S1**: Echocardiography before TAVR. TAVR = transcatheter aortic valve replacement.
