# Supplementary figures and images for: Transcatheter aortic valve replacement using the two-step inflation technique and the kissing-balloon technique for a patient with a protruding stent in the left main coronary artery: a case report
Source: Eur Heart J Case Rep. 2023 Nov 30;7(12):ytad575. doi: 10.1093/ehjcr/ytad575 (PMC10711422; doi:10.1093/ehjcr/ytad575)

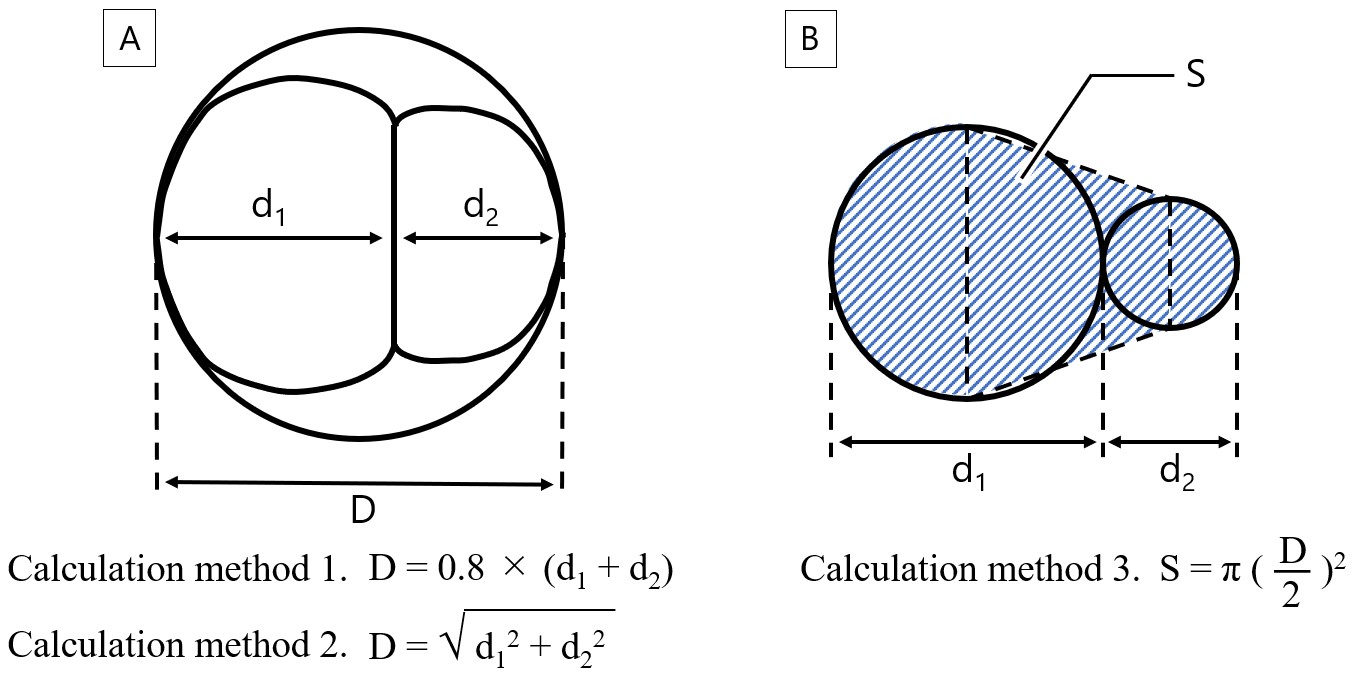

Supplement: ytad575_Supplementary_Data [file ytad575_supplementary_data.zip › Figure S1.jpg]
